# Scarcity of atrial fibrillation in a traditional African population: a community-based study

**DOI:** 10.1186/1471-2261-14-87

**Published:** 2014-07-18

**Authors:** Jacob JE Koopman, David van Bodegom, Rudi GJ Westendorp, Johan Wouter Jukema

**Affiliations:** 1 Department of Gerontology and Geriatrics, Postal zone C7-Q, Leiden University Medical Center, PO Box 9600, 2300 RC Leiden, the Netherlands; 2 Leyden Academy on Vitality and Ageing, 2333 AA Leiden, the Netherlands; 3 Department of Cardiology, Leiden University Medical Center, 2300 RC Leiden, the Netherlands

**Keywords:** Atrial fibrillation, Africa, Lifestyle, Inflammation, Electrocardiography, Aging

## Abstract

**Background:**

In western societies, atrial fibrillation is an increasingly common finding among the elderly. Established risk factors of atrial fibrillation include obesity, diabetes, hypertension, and cardiovascular disease. Atrial fibrillation has almost exclusively been studied in western populations where these risk factors are widely present. Therefore, we studied the epidemiology of atrial fibrillation in a traditional African community.

**Methods:**

In rural Ghana, among 924 individuals aged 50 years and older, we recorded electrocardiograms to detect atrial fibrillation. As established risk factors, we documented waist circumference, body mass index (BMI), capillary glucose level, blood pressure, and electrocardiographic myocardial infarction. In addition, we determined circulating levels of interleukin-6 (IL6), a proinflammatory cytokine, and C-reactive protein (CRP), a marker of systemic inflammation. We compared the risk factors with reference data from the USA.

**Results:**

Atrial fibrillation was detected in only three cases, equalling 0.3% (95% CI 0.1–1.0%). Waist circumference, BMI, and capillary glucose levels were very low. Hypertension and myocardial infarction were uncommon. Circulating levels of IL6 were similar, but those of CRP were lower compared with the USA.

**Conclusion:**

Atrial fibrillation is very scarce in this traditional African community. Its low prevalence compared with western societies can be explained by the rareness of its established risk factors, which are closely related to lifestyle, and by possible unmeasured differences in other risk factors or genetic factors.

## Background

Atrial fibrillation is a common finding among elderly in western societies. Its prevalence increases over age and mounts to 10 to 20% after the age of 80 years [1,2]. Due to ageing of western populations and improved survival from other cardiovascular disorders, the prevalence of atrial fibrillation has grown over time and is expected to continue growing. As it leads to a heightened risk of thromboembolism, cerebrovascular accidents, and congestive heart failure, its public health burden has grown concurrently [3,4].

The majority of the cases of atrial fibrillation can be attributed to the established risk factors obesity, diabetes, hypertension, prior cardiac disease, and smoking. Hypertension is the most important of these, responsible for at least one fifth of the cases [5,6]. Still, questions remain about the pathogenesis of atrial fibrillation. It is postulated that, next to these risk factors, inflammation plays an important role in the pathogenesis of atrial fibrillation, but this has not yet been confirmed [1,7]. Moreover, it is not clearly understood why atrial fibrillation is less commonly detected in black Americans, while they are more often affected by obesity, diabetes, and hypertension than white Americans [5,8,9]. However, the epidemiology of atrial fibrillation has been studied almost exclusively in western societies [1,3], where obesity, diabetes, hypertension, cardiovascular disease, and systemic inflammation are widely present among the elderly [10]. Little is known about the prevalence of atrial fibrillation in non-western societies, such as in rural Africa [11,12]. Knowledge about the risk of atrial fibrillation in the context of different environmental and genetic influences may provide more insight in its pathogenesis [13].

This study investigates the epidemiology of atrial fibrillation in a traditional rural African community where a sedentary lifestyle is absent. We used electrocardiography to detect atrial fibrillation among inhabitants aged 50 years and older. Established risk factors of atrial fibrillation have been documented, such as obesity, dysglycaemia, hypertension, and myocardial infarction. Circulating levels of interleukin-6 were measured as a marker of proinflammatory immune activation and circulating levels of C-reactive protein as a marker of systemic inflammation.

## Methods

### Research area

The Upper East Region is remote, rural, and one of the least developed regions of Ghana. The vast majority of the inhabitants is involved in non-commercial agriculture performed by manual labour [14]. The yearly per capita income averages US$ 135 [15]; 88% of the households lives in poverty [16]. Infectious diseases are the main causes of death [17].

Since 2002, we have registered and followed a traditional horticultural community in the Garu-Tempane District in the Upper East Region. This community occupies a research area of 375 km^2^ with approximately 25,000 inhabitants living in 32 villages. Migration is less than 1% per year [14,18,19]. Hospital care is absent; the nearest physician is at 40 kilometers’ distance. Sewage disposal systems are non-existent.

All inhabitants have been registered in a demographic database, including the name, age, sex, tribe, and household. We have determined the lifetime fertility, defined as the total number of children born per postreproductive woman, based on fertility data gathered in the research area in 2003 [19]. For each household, we have determined the household property value in 2007 according to the Demographic and Health Survey method [14]. In 2008, we have determined the prevalences of infections by malaria species by PCR of blood samples and those by helminths and protozoa by PCR of stool samples [20]. A more elaborate description of this cohort has been given elsewhere [14,18,19].

### Study population

Within the registered community, we aimed to estimate the prevalences of atrial fibrillation and its risk factors among individuals aged 50 years and older. For this, we set up a mobile field station in different villages during two field visits in 2009 and 2010. From here, all eligible inhabitants were approached. Inclusion was limited by the duration of the field visits. To ensure maximal participation and to avoid selective inclusion of healthy elderly, we brought less mobile participants by car. Of the approached inhabitants, 4.4% could not participate due to death since the last registration, 3.2% refused participation, 2.8% was absent from the research area during our visit because of migration or travelling, and 4.2% did not participate for other reasons. At the field work station, the identity of the participant was confirmed, the personal data in our registration were checked, and clinical and electrocardiographic investigations were performed. In addition, blood plasma samples were available of 266 individuals randomly selected across age groups in 2008. On these samples biochemical investigations were performed.

### Ethical approval

Ethical approval was given by the Ethical Review Committee of Ghana Health Service, the Committee Medical Ethics of the Leiden University Medical Center, and by the local chiefs and elders. Because of illiteracy, informed consent was obtained orally from the participants. A consent form with an explanation on the purpose and conduction of this research project was read out to each participant in his own language. The full text of the form was approved by the Ethical Review Committee of Ghana Health Service.

### Electrocardiographic investigations

A twelve-lead electrocardiogram was recorded twice for ten seconds in a lying and resting position (Schiller AT-104 PC). All electrocardiograms were assessed by an experienced cardiologist according to the Minnesota criteria. The cardiologist was blinded for the participants’ characteristics other than age and sex. A subset of the electrocardiograms (*n* = 610) was assessed for myocardial infarction [21]. The heart rate was described using both recordings. Bradycardia was defined as a sinus rhythm below 60/min on either recording. Tachycardia was defined as a sinus rhythm over 100/min on either recording. The electrocardiograms were classified as whether or not displaying atrial fibrillation or atrial flutter (codes 8-3-1, 8-3-2, 8-3-3, and 8-3-4).

### Clinical and biochemical investigations

We performed clinical investigations on those participating in the electrocardiographic investigations. We measured height, weight, waist circumference, glucose capillary blood concentration, and blood pressure. Body mass index (BMI) was calculated as weight divided by squared height (kg/m^2^). Hypertension was defined as a systolic blood pressure of 140 mmHg or higher and/or a diastolic blood pressure of 90 mmHg or higher [22].

We performed biochemical investigations on venous blood samples, collected in randomly selected individuals in 2008 (*n* = 266) [23] to measure circulating plasma levels of interleukin-6 (IL6) and C-reactive protein (CRP).

### Reference population

To compare our results with a western population, we derived data on the prevalences of the risk factors of atrial fibrillation from studies performed in the general population of the USA. Age and ethnicity-specific distributions of BMI and CRP and the prevalence of hypertension were derived from the National Health and Nutrition Examination Survey (NHANES) performed in 1999-2000 [24]. For the USA, the definition of hypertension was extended to include individuals using antihypertensive medication. Distributions of IL6 for white and black Americans of 65 years of age or older were derived from a publication by Cohen and colleagues [25].

These reference sources classified American ethnicities as white or black. Although they seem synonymous with Caucasian and African American ethnicities, we adopted their terminology for compatibility.

### Analyses

Prevalences of atrial fibrillation were calculated as the number of cases divided by the total number of inhabitants per ten-year age group and given as percentages. Prevalences of risk factors were given as percentages. Distributions of continuous variables were described by medians. Confidence intervals were calculated using Wilson’s formula for prevalences and using the binomial distribution for medians. Statistical analyses were performed with IBM SPSS Statistics version 20.

## Results

Table 1 provides a description of the demographic characteristics, the established risk factors of atrial fibrillation, and infectious and inflammatory markers for the Ghanaian study population aged 50 years and older. In the study population, the prevalences and levels of the established risk factors, including obesity, dysglycaemia, hypertension, and myocardial infarction, were very low. The levels of the inflammatory markers interleukin-6 (IL6) and C-reactive protein (CRP) are described. Infectious diseases were highly prevalent. These characteristics were similar between the entire registered population, the study population selected for the electrocardiographic and clinical investigations, and the subpopulation in which the biochemical investigations were performed, except for minor differences in the distributions of age and sex. The similarities remained after stratification by sex.

**Table 1:** General characteristics of the Ghanaian study population (age ≥ 50 years)

Individuals *n*	924	
Age *median (iqr) years*	66	(56–73)
Age groups *n (%)*		
50–59 years	307	(33.2)
60–69 years	291	(31.5)
70–79 years	242	(26.2)
80+ years	84	(9.1)
Male sex *n (%)*	480	(51.9)
Lifetime fertility *median (iqr) children per woman*	7	(6–9)
Households *n*	636	
Household property value *median (iqr) US$*	1,077	(533–1,942)
Waist circumference *median (iqr) cm*	76	(72–81)
Body mass index *median (iqr) kg/m*^*2*^	18.1	(16.5–19.5)
Capillary glucose *median (iqr) mmol/l*	3.9	(3.4–4.4)
Blood pressure *median (iqr) mmHg*		
diastolic	70	(65–80)
systolic	120	(110–135)
Hypertension	24.2	%
Myocardial infarction^a^	1.2	%
Individuals with infectious diseases^b^		
Malaria species	77.7	%
Protozoa	100.0	%
Helminths	21.5	%
Proinflammatory markers^c^		
Interleukin-6 *median (iqr) ng/l*	1.9	(1.4–2.7)
C-reactive protein *median (iqr) mg/l*	1.0	(0.4–2.7)

Lifetime fertility is expressed as the total number of children born per woman. Hypertension is defined as a systolic blood pressure of 140 mmHg or higher and/or a diastolic blood pressure of 90 mmHg or higher. Myocardial infarction represents electrocardiographically detected definite myocardial infarction. Circulating C-reactive protein (CRP) and interleukin-6 (IL6) have been measured in venous plasma samples. Iqr: interquartile range.

^a^Determined in a subpopulation of 610 individuals.

^b^Determined in 261 individuals for whom blood and stool samples were available.

^c^Determined in 266 individuals for whom plasma samples were available.

Electrocardiograms were obtained from 921 participants, of whom 479 males (52.0%). The median (interquartile) heart rate was 71 (63–80) per minute. Sinus bradycardia and tachycardia were present in 159 (17.3%) and 32 (3.5%) individuals. Three individuals had atrial fibrillation, equalling 0.3% (95% CI: 0.1–1.0%).

**Figure 1:**
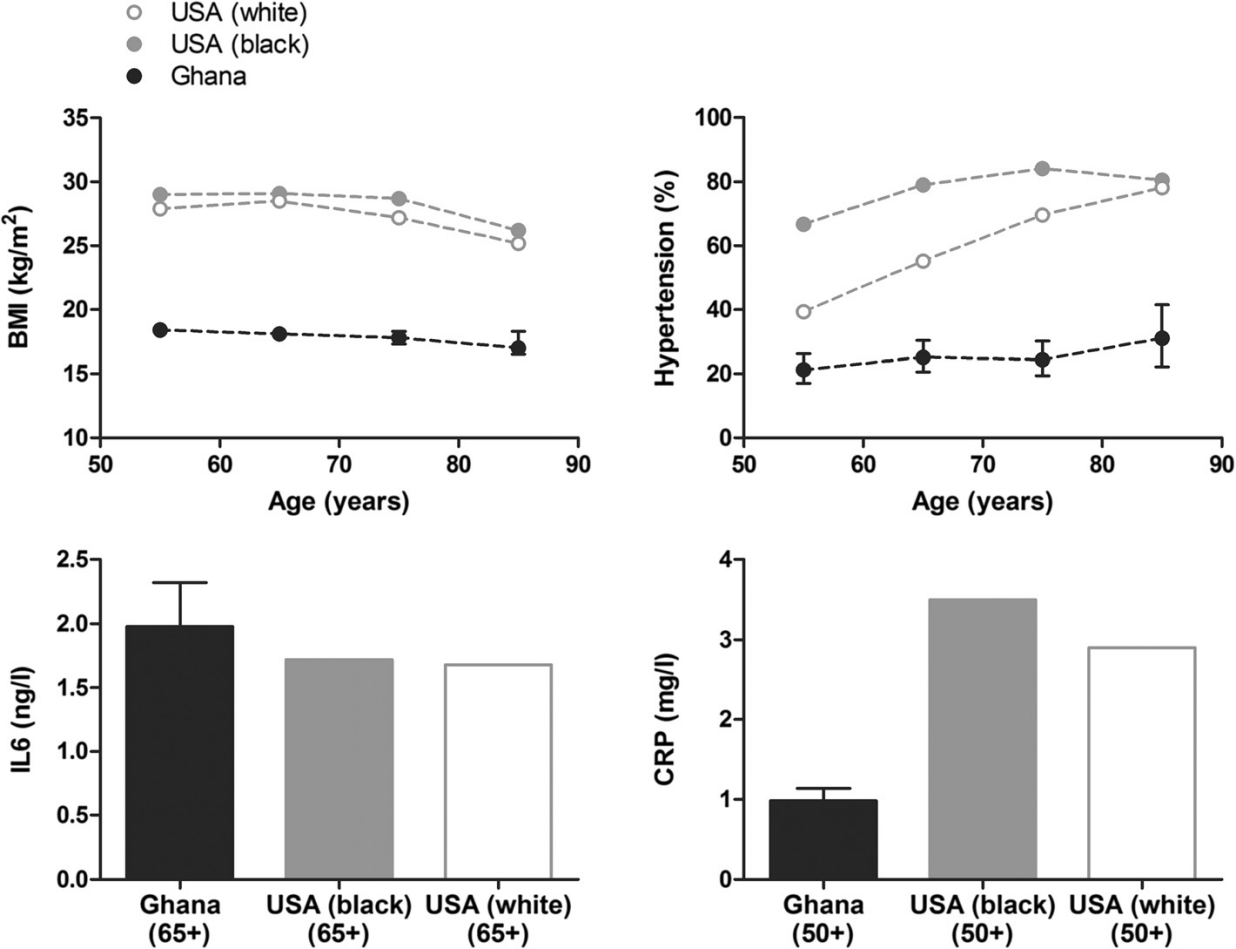
**Risk factors of atrial fibrillation in the Ghanaian study population and the USA.** As established risk factors, the distribution of body mass index (BMI) is given as median levels over age and prevalences of hypertension are given as percentages over age. Hypertension is defined as a systolic blood pressure of 140 mmHg or higher and/or a diastolic blood pressure of 90 mmHg or higher, and, for the USA, treatment with antihypertensive medication. As proinflammatory markers, distributions of interleukin-6 (IL6) and C-reactive protein (CRP) are given as median levels over the age of 65 and 50 years, respectively. For the Ghanaian study population, 95% confidence intervals are given. As references, prevalences are given for white and black ethnicities in the general population of the USA. These data have been derived from NHANES 1999-2000 [24] and from Cohen and colleagues [25].

Table 2 shows characteristics of the three cases with atrial fibrillation in the Ghanaian study population. Of these three, one was female and two were male. The first two were affected by hypertension. The third case was a smoker. Only for the latter case inflammatory markers were known; compared with the entire study population as well as the USA, these levels were low for both IL6 and CRP.Figure 1 compares the distributions over age of the most important established risk factors of atrial fibrillation and compares the levels of the inflammatory markers with known distributions and levels in the USA, separated for white and black ethnicities. The levels of BMI and hypertension in the Ghanaian study population were lower than those of both white and black Americans. The levels of IL6 were comparable with, but the levels of CRP were lower than those of both white and black Americans.

**Table 2:** Characteristics of the three cases with atrial fibrillation in the Ghanaian study population

**Case**	**Sex**	**Age**	**HR**	**RR**	**BMI**	**Gluc**	**Smok**	**Isch**	**Infa**	**SES**
**1**	♀	76	104	140/85	21.8	3.2	–	–	–	420
**2**	♂	76	80	165/80	17.5	3.1	–	–	–	530
**3**	♂	76	104	125/80	19.1	3.8	+	–	–	1,030

HR: ventricular heart rate (/min). RR: systolic/diastolic blood pressure (mmHg). BMI: body mass index (kg/m^2^). Smok: smoking. Gluc: capillary blood glucose concentration (mmol/l). Isch: electrocardiographic myocardial ischaemia-like repolarisation abnormalities. Infa: electrocardiographic infarction. SES: household value (US$). Smok, Isch, and Infa, are designated as present (+) or absent (–).

## Discussion

In this study we showed that atrial fibrillation was very scarce after the age of 50 years in a traditional rural community in Africa. The near absence of atrial fibrillation in the Ghanaian study population confirms the low prevalences that have been found by a few studies in other traditional African populations. In rural Tanzanians aged 70 years and older, its prevalence was 0.7% [11]. In the South African Bantu population, atrial fibrillation was detected in 0.2% of patients attending a cardiac clinic but not diagnosed with cardiac disease [26]. In patients from the Bantu population hospitalised because of cardiac failure, it was present in 12% [27].

The prevalence of atrial fibrillation in urban African populations is higher than those in rural African populations. In a South-African study covering both urban and rural communities, atrial fibrillation was detected in 2% of blacks over the age of 30 years [28]. In two large cardiologic hospitals, 4.6% and 5.5% of the admitted patients had atrial fibrillation at relatively young ages [29,30]. Among cardiologic hospitals across several Sub-Saharan African countries, atrial fibrillation was found in 18% of cases with acute heart failure [31].

The prevalence of atrial fibrillation in western populations is higher than those in rural African populations. Several studies in patient populations and the general populations of the USA and Western Europe have reported its prevalence to rise from less than 2% around the age of 50 years up to 10 to 20% after the age of 80 years [1]. In the general population of the USA, similar increases over age have been described for both white and black Americans [2].

The low prevalence of atrial fibrillation in rural African populations compared with urban African and western populations can be explained by a similarly lower prevalence of its established risk factors, including obesity, diabetes, hypertension, and cardiovascular disease. These risk factors are closely related to a sedentary lifestyle [5,10]. With the transition, urbanisation, and ageing of African populations, a sedentary lifestyle is adopted and the prevalence of atrial fibrillation rises [12,30].

Interestingly, cases of atrial fibrillation described in African populations are accompanied by underlying cardiac disorders in proportions up to 90%, which contrasts with the large proportion of idiopathic cases described in western populations [3,4]. Mostly, these cardiac disorders concern hypertensive cardiopathy and rheumatic valvular heart disease [12,27,29,30]. In the Ghanaian study population, two of the three cases suffered from hypertension. This relationship between cardiac disease and atrial fibrillation supports that atrial fibrillation may be mainly propagated by obesity, diabetes, hypertension, and cardiovascular disease.

Recently, inflammation has been postulated to play an important role in the pathogenesis of atrial fibrillation [7]. In the Ghanaian study population the level of IL6, an instigator of a proinflammatory response, was similar to that in the general population of the USA. Earlier we have shown that the study population is biochemically and genetically enriched with proinflammatory markers, probably due to the endemic high infectious load [23,32,33]. On the other hand, the level of CRP, a marker of systemic inflammation, was lower compared with the USA. Similarly, we have previously reported that the median level of CRP as well as the prevalence of mildly elevated levels of CRP was lower in the study population compared with the general population in the Netherlands. This difference was attributable to a lower BMI in the Ghanaian study population [34]. Together, these findings may indicate that, while the capacity to generate an inflammatory response is preserved, systemic inflammation is uncommon in the Ghanaian study population.

Mendelian randomisation has shown that an elevation of CRP is rather an effect than a cause of atrial fibrillation [35]. This interpretation is supported by observations that deny an association between CRP level and history of atrial fibrillation, but confirm that CRP is elevated during episodes of atrial fibrillation or due to coexistence of hypertension or obesity [36-38]. Inflammatory processes related to atrial fibrillation seem to be caused by ischaemic or oxidative injury of atrial myocytes, which is again caused by obesity, diabetes, hypertension, and cardiac disease [39,40]. In the Ghanaian study population, hypertension is present in only about a quarter and obesity, dysglycaemia, and cardiovascular disease are rare. The low levels of CRP match with the close relation between these risk factors and inflammation. We have observed a similar pattern when studying inflammation in relation to peripheral and coronary arterial disease in this study population [21].

While we found almost no atrial fibrillation in the Ghanaian study population, hypertension was present in a considerable proportion. Similarly, in western populations, black ethnicities are more affected by obesity, diabetes, and hypertension, but less often develop atrial fibrillation compared with white ethnicities [5,8,11]. Meanwhile, the associations between these risk factors and atrial fibrillation are similar in both ethnicities [5,9,41,42]. A solution of this paradox may be provided by the higher sensitivity that seems required for methods to detect atrial fibrillation in blacks compared with whites [43]. However, more research in different populations is needed to unravel the interactions between environmental and genetic risk factors [13].

The low prevalence of atrial fibrillation in the Ghanaian study population may also be a result of a lack of risk factors other than those measured in this study. No data was available on the prevalence of rheumatic heart disease, which is a common cause of atrial fibrillation in African populations [12,29,30]. Furthermore, we had no information on thyroid disease, smoking, and alcohol use, which are risk factors of atrial fibrillation in western populations [3,4].

Differences between ethnicities in the risks of atrial fibrillation are possibly caused by genetic factors. Multiple genetic polymorphisms have been associated with an elevated risk of atrial fibrillation, mainly in studies on populations from European decent. Yet, the effects of these associations are modest and differ between ethnicities [44,45]. The lower prevalence of atrial fibrillation among black Americans compared with white Americans may be a result of a lower frequency of genetic variants that predispose to atrial fibrillation among blacks. As black Americans show great genetic similarity with populations in West Africa, where the Ghanaian study population is located [46], a lower frequency of such genetic variants may likewise explain why atrial fibrillation was scarce in the Ghanaian study population. However, this remains speculative: one report states that European genetic admixture does not explain the differences in prevalence of atrial fibrillation between white and black Americans [47], while another report contradicts this [48]. Moreover, if the frequency of genetic variants that predispose to atrial fibrillation would be lower among black Americans, the greater prevalence of atrial fibrillation among black Americans compared with the Ghanaian study population reinforces the essential role of lifestyle-related factors rather than such genetic variants.

The scarcity of atrial fibrillation in our study population can also be explained by selective survival of unaffected individuals. When sufficient medical care is absent, patients with atrial fibrillation may decease early from the underlying disorders or complications, such as cardiac disease or stroke. A study on patients with atrial fibrillation in rural Tanzania reports that 8 out of 15 died within a year after detection [11]. In a larger group in Cameroon one-year mortality was 30%, of which more than half was of cardiovascular origin. Of the survivors, 18% experienced cerebrovascular accidents [12]. On the other hand, with relatively low risk scores [12,30] atrial fibrillation in these populations seems unlikely to be so severely lethal to render infinitesimal prevalence estimates. In line with this, we have determined in another study [49], by means of verbal autopsy on 1,263 of the 1,406 deaths that were registered in our cohort population, that only 2.7% died from cardiovascular causes. For those who died at the age of 50 years or older, this was 4.5%.

This study on atrial fibrillation in a traditional rural African population has limitations. First, the use of two subsequent electrocardiographic recordings of ten seconds may be insufficient to detect all cases of atrial fibrillation. More elaborate screening techniques used in western populations yield more reliable estimates of its prevalence; this difference in methodology hampers the comparison of our results with data from western populations. Second, the documentation of the cardiovascular risk factors lacks information on history and family history of cardiovascular disease. Third, due to different life expectancies, the number of elderly aged 50 years and older is lower in our population than in western populations. Fourth, due to the cross-sectional nature of this study, presence or absence of causality in the relationships between the established risk factors, systemic inflammation, and atrial fibrillation cannot be demonstrated.

The data on atrial fibrillation and its risk factors in the Ghanaian study population are not necessarily generalisable to other African populations, as they vary genetically and culturally [46]. In the same manner, the comparison between the Ghanaian study population and the general population of the USA may not reflect a universal difference between African and western populations. Comparisons between divergent populations are informative, but also complicated. As described above, some possible differences between the Ghanaian study population and the general population of the USA remain largely unknown. More research in different non-western populations is needed to overcome the limitations of this study and to extend the scarce knowledge on atrial fibrillation in such populations [13].

## Conclusion

In conclusion, we have shown that atrial fibrillation is nearly absent in a traditional rural community in Ghana. The low prevalence compared with western societies can be explained by the rareness of its established risk factors, including obesity, diabetes, hypertension, and cardiac disease, as well as systemic inflammation, which are closely related to a sedentary lifestyle and uncommon in this population. Future research is needed to elucidate the roles of other risk factors and genetic factors.

## Competing interests

The authors declare that they have no competing interests.

## Authors’ contributions

JJEK and DvB have performed the experiments. JWJ has delivered analysis tools. JJEK and JWJ have analysed the data. JJEK and DvB have written the manuscript. All authors have conceived and designed the experiments, have interpreted the data, and have provided intellectual input for the manuscript. All authors have read and approved the final manuscript.

## Pre-publication history

The pre-publication history for this paper can be accessed here:

http://www.biomedcentral.com/1471-2261/14/87/prepub
